# Increased midlife triglycerides predict brain β-amyloid and tau pathology 20 years later

**DOI:** 10.1212/WNL.0000000000004749

**Published:** 2018-01-02

**Authors:** Katarina Nägga, Anna-Märta Gustavsson, Erik Stomrud, Daniel Lindqvist, Danielle van Westen, Kaj Blennow, Henrik Zetterberg, Olle Melander, Oskar Hansson

**Affiliations:** From the Clinical Memory Research Unit (K.N., A.-M.G., E.S., O.H.) and Clinical Research Centre (O.M.), Department of Clinical Sciences Malmö, Lund University; Memory Clinic (K.N., A.-M.G., E.S., O.H.), Skåne University Hospital, Malmö; Psychiatry (D.L.) and Diagnostic Radiology (D.v.W.), Department of Clinical Sciences Lund, Lund University; Department of Psychiatry and Neurochemistry (K.B., H.Z.), Institute of Neuroscience and Physiology, The Sahlgrenska Academy at University of Gothenburg; Clinical Neurochemistry Laboratory (K.B., H.Z.), Sahlgrenska University Hospital, Mölndal, Sweden; and Department of Molecular Neuroscience (H.Z.), UCL Institute of Neurology, Queen Square, London, UK.

## Abstract

**Objective:**

To evaluate the effect of midlife lipid levels on Alzheimer brain pathology 20 years later in cognitively normal elderly individuals.

**Methods:**

This is a longitudinal cohort study of 318 cognitively normal individuals with data on fasting lipid levels at midlife (mean age 54 years). Presence of β-amyloid (Aβ) and tau pathologies 20 years later (mean age 73 years) were detected by quantifying Alzheimer disease (AD) biomarkers in CSF. In a subset (n = 134), Aβ (^18^F-flutemetamol) PET was also performed.

**Results:**

CSF Aβ_42_ and Aβ PET revealed Aβ pathology in approximately 20% of the cognitively healthy population and CSF Aβ_42_/phosphorylated tau (p-tau) ratio indicated both Aβ and tau pathology in 16%. Higher levels of triglycerides in midlife were independently associated with abnormal CSF Aβ_42_ (odds ratio [OR] 1.34, 95% confidence interval [CI] 1.03–1.75, *p* = 0.029) and abnormal Aβ_42_/p-tau ratio (OR 1.46, 95% CI 1.10–1.93; *p* = 0.009) adjusting for age, sex, *APOE* ε4, education, and multiple vascular risk factors. Triglycerides were also associated with abnormal Aβ PET in multivariable regression models, but the association was attenuated in the fully adjusted model. Increased levels of medium and large low-density lipoprotein subfractions were significantly associated with abnormal Aβ PET and large high-density lipoprotein particles were associated with decreased risk of abnormal Aβ PET.

**Conclusions:**

Increased levels of triglycerides at midlife predict brain Aβ and tau pathology 20 years later in cognitively healthy individuals. Certain lipoprotein subfractions may also be risk factors for Aβ pathology. These findings further support an involvement of lipids in the very early stages of AD development.

Genetic analyses indicate that lipid metabolism is one of the main pathways involved in the pathologic process of Alzheimer disease (AD),^1^ but previous longitudinal cohort studies show inconsistent results regarding lipids and later development of AD dementia.^2^ Factors explaining these discrepancies include varying follow-up times^2^ and the fact that a clinical diagnosis of AD is often unreliable.^3^ Autopsy studies assessing the longitudinal relationship between lipid levels and brain AD pathology include individuals with varying cognitive status.^4,5^ Since cerebral β-amyloid (Aβ) accumulation seems to start 10–20 years before symptom onset,^6^ cognitively normal individuals should be studied when determining risk factors associated with the earliest AD events. Aβ accumulation is detected reliably using either Aβ PET or CSF biomarkers.^7^ One previous cross-sectional study in healthy elderly found an association between higher triglycerides and Aβ PET^8^ and 2 recent longitudinal studies showed that an increased number of midlife vascular risk factors^9^ and midlife dyslipidemia^10^ were associated with brain amyloid deposition measured with PET. We aimed to investigate the association between different lipid levels in midlife and presence of brain amyloid 20 years later in asymptomatic individuals who were cognitively normal at follow-up. Further, we aimed to study if lipoprotein subfractions are associated with AD pathology. Previous findings suggest that high-density lipoprotein (HDL) and low-density lipoprotein (LDL) are independent risk factors for cardiovascular disease and may be superior to conventional HDL and LDL in assessing cardiovascular risk.^11–14^ Their role as risk factors for AD has not been studied previously.

## Methods

### Participants

The present data are derived from the cognitively healthy cohort of the Swedish BioFINDER Study (biofinder.se). In short, participants were recruited from the longitudinal population-based Malmö Diet and Cancer Study (MDCS) cardiovascular cohort (previously described in more detail).^15–17^ The baseline examination was performed between 1991 and 1994, followed by a reinvestigation between 2007 and 2012. Seventy-six percent of the eligible baseline population attended the reinvestigation and dropouts were generally in poorer health condition than attendees.^17^ Reasons for nonparticipation were unwillingness, sickness, or lack of information in registers. At baseline, all participants responded to questionnaires on health status and medication use and underwent physical examinations (anthropometric measures) and blood sampling by trained research nurses.^15,17,18^

Beginning in 2009, we recruited candidates to the cognitively healthy cohort from the ongoing MDCS reinvestigation based on the following criteria: age >60 years, Mini-Mental State Examination (MMSE) score ≥27 points, and no subjective cognitive impairment. During random periods, individuals fulfilling these criteria were invited to participate in the cognitively healthy cohort of the Swedish BioFINDER Study. This resulted in 437 potential study participants, who underwent a thorough clinical examination at the Memory Clinic by trained medical doctors assessing neurologic and psychiatric status as well as cognitive function (including a second MMSE), described previously in more detail.^19^ Individuals failing to fulfill inclusion criteria after this refined assessment, or with a history of TIA or stroke, severe neurologic or psychiatric disease, dementia, or mild cognitive impairment (Clinical Dementia Rating score >0), were excluded. Based on these criteria, 76 individuals were excluded, which yielded 361 participants in the cognitively normal cohort of the Swedish BioFINDER Study. The inclusion was terminated when this predefined number of participants was reached. Between 2010 and 2015, participants underwent cognitive testing,^19^ brain MRI, and lumbar puncture. After study completion, 318 individuals had available data on CSF biomarkers and midlife laboratory tests, and were included in the present study.

### Standard protocol approvals, registrations, and patient consents

All individuals received information about the study and gave written consent to participate. The Ethical Committee of Lund University, Lund, Sweden, gave ethical approval.

### CSF analyses

The procedure and analysis of the CSF followed the Alzheimer's Association Flow Chart for CSF biomarkers.^20^ We collected lumbar CSF samples between 2010 and 2015 and later analyzed them simultaneously according to a standardized protocol.^21^ CSF concentrations of Aβ_42_ and tau phosphorylated at Thr181 (p-tau) were measured with INNOTEST ELISA (Fujirebio Europe, Ghent, Belgium).

### Aβ PET imaging

Brain Aβ was also measured using [^18^F]flutemetamol PET^22^ in a subpopulation (n = 134). We conducted PET/CT scanning between 2013 and 2015 using the same type of Philips (Best, the Netherlands) Gemini TF 16 scanner at 2 different sites. Average uptake was estimated from PET sum images from 90 to 110 minutes after injection. The images were analyzed with the NeuroMarQ software provided by GE Healthcare (Cleveland, OH). A volume of interest template was applied for 9 bilateral regions (prefrontal, parietal, lateral temporal, medial temporal, sensorimotor, occipital, anterior cingulate, and posterior cingulate/precuneus), combined in a global neocortical composite signal.^23^ The standardized uptake value ratio (SUVR) was the global composite tracer uptake, normalized for the mean uptake in the cerebellar cortex.

### MRI of white matter lesions

We examined 308 participants between 2009 and 2015 using a 3T MRI scanner (Trio; Siemens, Munich, Germany). The MRI protocol comprised axial T2 fluid-attenuated inversion recovery (FLAIR) imaging and a coronal magnetization-prepared rapid gradient echo (MPRAGE) sequence. The total white matter lesion (WML) volume (mL) was segmented from the MPRAGE and FLAIR image data using the Lesion Segmentation Tool, version 1.2.3, as implemented in SPM8.^24^

### Lipid analyses

Triglycerides, cholesterol, and HDL were measured in serum after an overnight fast at the baseline visit in MDCS (1991–1994) using standard clinical procedures. LDL was calculated using the Friedewald formula.^18^

For quantification of plasma lipoprotein subfractions, lipoproteins were isolated by dextran sulfate precipitation as previously described.^13,14^ The lipoproteins were fractionated and quantitated in a single scan using gas-phase electrophoresis (ion mobility).^25^ This method separates and quantitates lipoprotein particles ranging in size from small HDL to large very low-density lipoproteins (VLDLs).

### Covariates

Covariates were selected based on available study data and on previous literature, linking vascular risk factors to AD.^26^ *APOE* ε4 status was introduced as the absence or presence of any ε4 allele in order to investigate whether associations were independent of this well-known genetic risk factor for AD. We collected data on years of education at inclusion in the Swedish BioFINDER Study. All other covariates were derived from the baseline visit in MDCS (1991–1994) and details on data collection have been described previously.^15,17,18^ Mean intima-media thickness (IMT), a surrogate marker for subclinical atherosclerosis,^27^ was assessed according to standard procedures in the right common carotid artery.^17^ Systolic and diastolic blood pressure was measured after 10 minutes of resting in a supine position. Fasting blood glucose was measured after an overnight fast using standard clinical procedure.^18^ We calculated body mass index as kg/m^2^. Cardiovascular disease, smoking, physical activity, and lipid-lowering medication were self-reported data derived from the self-administered questionnaire (http://links.lww.com/WNL/A48),^15,16^ described in more detail in e-Methods (http://links.lww.com/WNL/A17).

### Statistical analysis

We used SPSS (SPSS Inc., Chicago, IL) statistical software (version 22 for Windows) for all statistical analyses, except mixture modeling, which we performed using R (version 3.1.2 with Mixtools version 1.0.2). CSF and PET data were dichotomized and used as dependent variables in logistic regression models. Cutoff values for CSF and PET data were estimated using mixture modeling where a bimodal distribution reveals a cutoff point.^21^ The estimated cutoffs were abnormal Aβ_42_ <500 pg/mL, abnormal Aβ_42_/p-tau ratio <7.7, and abnormal PET composite SUVR >1.42. We assessed WML volume as a continuous dependent variable using linear regression models.

We performed multivariable regression models with backward elimination (removal at *p* > 0.1) and constructed 4 models adding covariates in a stepwise manner as follows: model 1: age; model 2: age, sex, *APOE* ε4, and education; model 3: age, sex, *APOE* ε4, education, IMT, systolic blood pressure, fasting blood glucose, and body mass index; model 4: age, sex, *APOE* ε4, education, IMT, systolic blood pressure, fasting blood glucose, body mass index, cardiovascular disease, smoking, physical activity, and lipid-lowering medication (at follow-up). A *p* value <0.05 was considered statistically significant. Linear data were converted to *Z* scores, using score (x), mean (μ), and SD (σ) according to the formula z = (x − μ)/σ. In the multivariable analyses, only participants with data on all entered covariates were included in the analyses.

We also performed sensitivity analyses regarding blood pressure, weight reduction, and lipid-lowering medication at baseline and tested statistical interaction between triglycerides and *APOE* ε4 (presented in e-Results, http://links.lww.com/WNL/A17).

## Results

Table 1 presents characteristics of the cognitively normal cohort of the Swedish BioFINDER Study in comparison with the recruitment cohort. The study group was generally healthier than the other MDCS participants (table 1). Mean follow-up time between the baseline visit and subsequent lumbar puncture was 20 years (SD ± 1.6) and mean follow-up time between baseline visit and PET imaging was 21 years (SD ± 0.9). CSF and PET biomarkers revealed abnormal amounts of Aβ in approximately 20% of the cognitively healthy population and indicated a combination of Aβ and tau pathology in 16%.

**Table 1:**
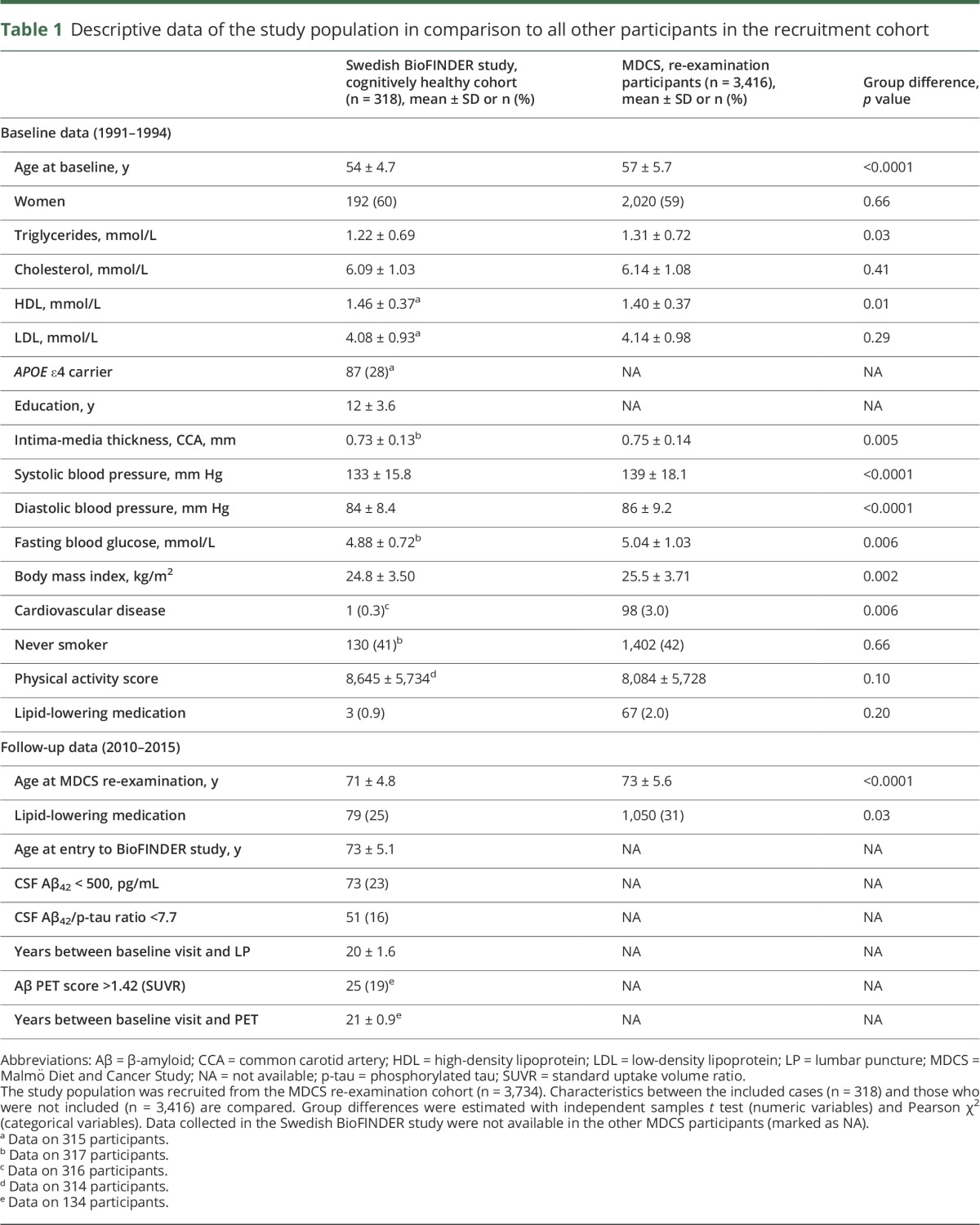
Descriptive data of the study population in comparison to all other participants in the recruitment cohort

### Baseline lipid levels and abnormal CSF biomarkers 20 years later

Associations between lipid levels (obtained at baseline in 1991–1994) and AD pathology 20 years later are presented in table 2. Higher levels of triglycerides in midlife were associated with abnormal CSF Aβ_42_, as well as abnormal CSF Aβ_42_/p-tau ratio, in all logistic regression models even after multivariable adjustments (table 2).

**Table 2:**
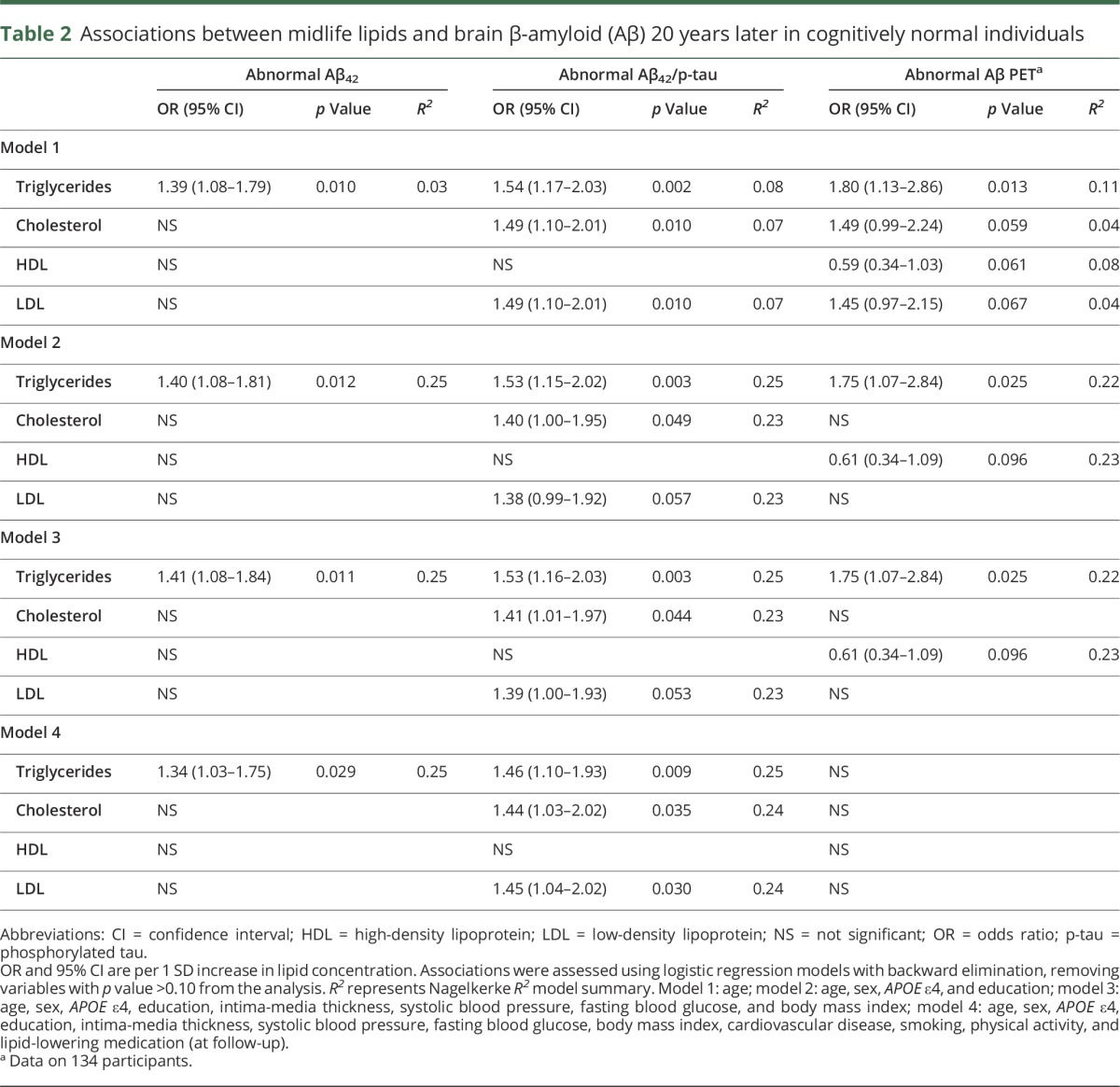
Associations between midlife lipids and brain β-amyloid (Aβ) 20 years later in cognitively normal individuals

Total cholesterol was associated with Aβ_42_/p-tau ratio in all logistic regression models (table 2). LDL was also associated with abnormal Aβ_42_/p-tau ratio in models 1 and 4, but the significance level was somewhat attenuated in models 2 and 3 (table 2). HDL was not associated with the measured CSF biomarkers (table 2).

### Baseline lipid levels and abnormal Aβ PET imaging 21 years later

In the subpopulation with available Aβ PET data (n = 134), higher triglyceride levels were associated with abnormal Aβ PET 21 years later in models 1 through 3 (table 2). The association was attenuated and did not reach significance in model 4. Cholesterol, HDL, and LDL were not significantly associated with abnormal Aβ PET (table 2).

### Baseline lipoprotein subfractions and abnormal Aβ PET imaging 21 years later

We further analyzed different lipoprotein subfractions in blood (n = 117) and found that medium and large LDL were significantly associated with abnormal Aβ PET in multivariable regression models (table 3). Large HDL indicated decreased risk of Aβ pathology (table 3). Small HDL and very small LDL were associated with increased risk in models 1 through 3, but not in model 4 (table 3). Intermediate-density lipoproteins and VLDL particles were not associated with Aβ PET (table 3).

**Table 3:**
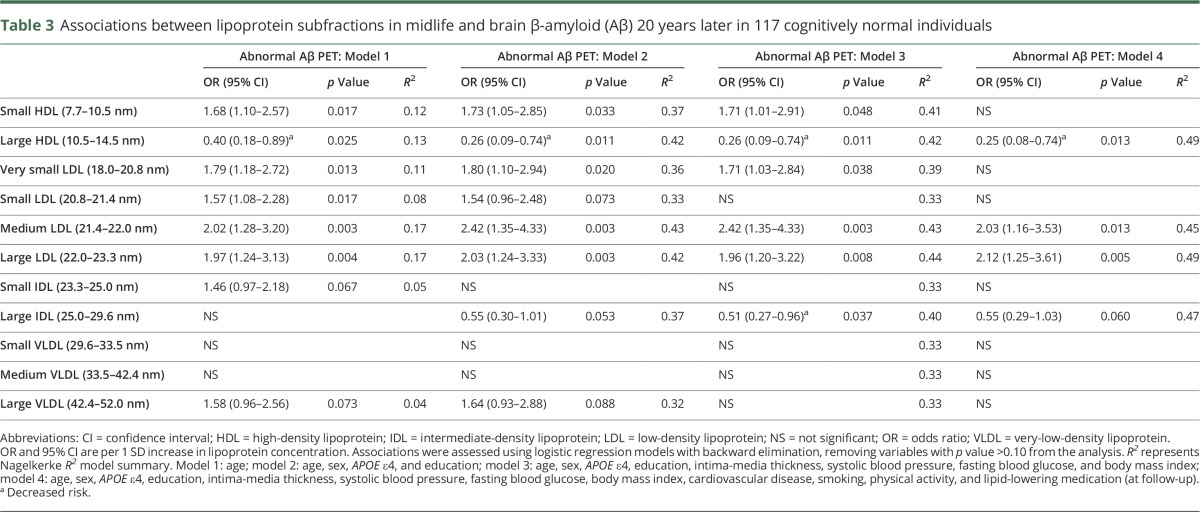
Associations between lipoprotein subfractions in midlife and brain β-amyloid (Aβ) 20 years later in 117 cognitively normal individuals

### Baseline lipid levels and WMLs in the brain 20 years later

Next, we analyzed the associations between midlife lipid levels and the presence of small-vessel brain disease quantified as WML volume using MRI. None of the measured lipids was associated with WML volume in the multivariable linear regression models (*p* > 0.05).

## Discussion

In this longitudinal study of 318 elderly individuals with normal cognition, we found that higher fasting triglyceride levels in midlife were associated with increased risk of brain Aβ and tau pathology 20 years later. This finding was independent of age, sex, *APOE* ε4, and vascular risk factors. In the subpopulation with available Aβ PET (n = 134), the association was attenuated and did not reach significance in model 4, where self-reported variables were added to the analyses (table 2). Most previous studies assessing lipid levels and Aβ accumulation were cross-sectional^8,28–30^ and longitudinal study designs are essential in order to assess causality and to evaluate mechanisms that influence pathologic processes in the early stages.

Interestingly, in a recently published longitudinal study, only dyslipidemia, among other midlife vascular risk factors, was associated with cerebral Aβ deposition.^10^ Another recent study showed that a cumulative number of midlife vascular risk factors, including cholesterol levels, was associated with Aβ PET.^9^ These studies did not report triglyceride levels. Longitudinal data from autopsy studies also suggest that dyslipidemia may increase the risk of neuritic plaques, but the results are inconsistent and over one-third of the participants in these studies had a dementia diagnosis.^4,5^ Since amyloid deposition begins 10–20 years before the onset of cognitive symptoms,^6^ we specifically aimed to study individuals who had not yet reached a state of cognitive impairment.

Several large studies indicate that increased midlife cholesterol level is associated with a clinical diagnosis of AD and dementia.^31–33^ However, it is still not completely understood how dyslipidemia exerts its pathologic effect on AD development.^34^ To identify the processes involved in accumulation and deposition of Aβ, early identification of disease-modifying mechanisms may be necessary, and therefore studies on presymptomatic stages of AD are important. In our study, midlife cholesterol was associated with CSF Aβ_42_/p-tau ratio, which is a measure of combined Aβ and tau pathology and may indicate more advanced AD pathology than CSF Aβ_42_ alone or Aβ PET. Even though our results were not robust regarding all Aβ measures, our findings together with previous studies^31–33^ may indicate that triglycerides are associated with early Aβ accumulation and cholesterol is associated with later stages of the predementia phase of AD.

The potential pathophysiologic effect of triglycerides on Aβ pathology is unclear. Plasma triglyceride levels have been shown to be increased prior to Aβ deposition in transgenic AD mouse models, pointing at a direct association between triglycerides and Aβ homeostasis.^35^ Lipids may influence membrane fluidity, which could directly affect secretase-mediated Aβ.^36^ In vitro data also suggest that lipids may have a direct effect on Aβ aggregation kinetics.^37^ Finally, triglyceride-rich lipoprotein particles in blood may serve as Aβ carriers.^38^ Thus, there are a multitude of potential pathways through which midlife lipid concentrations could influence the risk of developing cerebral β-amyloidosis and clinical AD, which warrants further research.

We found no associations between midlife lipid levels and WML load in our study, which may indicate that the association between triglycerides and Aβ is not mediated through hyperlipidemia being a vascular risk factor in general. Neither did fasting blood glucose, as a measure of diabetic or prediabetic status, attenuate the association between triglycerides and AD pathology. In any case, our data, along with data derived from animal models,^35^ suggest a direct relationship between triglycerides and AD.

There is evidence indicating that lifestyle interventions lower triglyceride levels through physical activity, diet control, and weight reduction. Pharmacologic treatments with triglyceride-lowering effects include statins, fibrates, niacin, and N-3 fatty acids.^39,40^ So far, these treatments have mainly been tested to reduce the risk of cardiovascular disease. If our finding that increased triglyceride levels in midlife lead to an increase in abnormal Aβ accumulation can be reproduced in larger cohorts, it would be of great interest to initiate intervention trials with triglyceride-lowering therapies in midlife and study potential long-term reductions in Aβ as the main outcome. If such treatments can decrease the risk of developing AD pathology, this could lead to significant health improvements for millions of people.

Apart from the standard lipid analyses, different lipoprotein subfractions may add new insights into the risk profiles of blood lipids.^11^ Because lipoproteins are heterogeneous in structure and function,^13,14^ the different subfractions may have distinctive characteristics in relation to AD development. In our study, higher levels of medium and large LDL subfractions, but lower levels of large HDL, were associated with Aβ pathology. Since neither standard HDL nor LDL were associated with AD pathology in the study population, this may point towards an added value of measuring lipoprotein subfractions in AD risk assessments. However, these results are based on a smaller population and are mainly explorative, and presented in order to encourage further investigation. In previous studies, small HDL particles have been shown to increase the risk of cardiovascular disease, whereas larger subfractions of HDL seem to be protective.^12^ Interestingly, our findings indicate the same pattern of association with Aβ as outcome. The effects of the different LDL subfractions seem to be more diverse. One study found that small- and medium-sized fractions of LDL have the most atherogenic effect,^14^ whereas another study did not find that the smallest LDL fractions were associated with increased risk of cardiovascular disease.^13^ Since both cardiovascular disease and AD seem to be attributable to the same vascular risk factors, continued investigation of lipoprotein subfractions is of interest.

This study has potential limitations. Since we recruited our cohort at the MDCS re-examination, only attendees were eligible for inclusion. The participants included in the present study were healthier than the majority of MDCS re-examination attendees (table 1). This introduces selection bias, where study participants are healthier than the general population, which may lead to an underestimation of the found associations. The results need to be replicated in other and preferably larger cohorts, in order to establish the association between midlife triglycerides and subsequent Aβ accumulation. However, our sample size was considerably larger than in previous studies assessing lipids and brain Aβ.^4,5,8,28–30^

Although we were able to adjust for many demographic factors, not all possible confounders could be addressed. However, we included the same control variables as previous studies on lipids and brain Aβ, generally adjusting for age, sex, *APOE* ε4, lipid-lowering treatment, and vascular risk factors.^8,28–30^ Cardiovascular disease was self-reported, which possibly underestimates the true prevalence and may be less reliable than other measures, e.g., medical diagnoses. We tried to compensate this by using other objective measures of cardiovascular pathologies, such as IMT, which is a marker for atherosclerosis.^27^

Since we aimed to explore the potential added value of lipoprotein subfractions apart from standard lipid analyses, multiple testing was performed. This may lead to findings mainly due to chance, and consequently our results regarding subfractions ought to be interpreted with caution. Another weakness is that lipid levels were only measured on one occasion in midlife, but this approach is common in population-based settings.

Despite these limitations, the current study contributes valuable new information. Strengths of the study include a long follow-up period together with thorough adjustments for both vascular risk factors and *APOE* ε4 carrier status, as well as direct analyses of Aβ pathology using both CSF and PET. If our findings can be replicated, increased triglycerides may be recognized as a modifiable and easily measured risk factor for AD pathology.

## Glossary

Aβ: β-amyloid
AD: Alzheimer disease
FLAIR: fluid-attenuated inversion recovery
HDL: high-density lipoprotein
IMT: intima-media thickness
LDL: low-density lipoprotein
MDCS: Malmö Diet and Cancer Study
MMSE: Mini-Mental State Examination
MPRAGE: magnetization-prepared rapid gradient echo
p-tau: tau phosphorylated at Thr181
SUVR: standardized uptake value ratio
VLDL: very low-density lipoprotein
WML: white matter lesion

## References

[1] Guerreiro R, Hardy J. Genetics of Alzheimer's disease. Neurotherapeutics 2014;11:732–737. 25113539 10.1007/s13311-014-0295-9 PMC4362699

[2] Reitz C. Dyslipidemia and the risk of Alzheimer's disease. Curr Atheroscler Rep 2013;15:307. 23328907 10.1007/s11883-012-0307-3 PMC3564220

[3] Brunnstrom H, Englund E. Clinicopathological concordance in dementia diagnostics. Am J Geriatr Psychiatry 2009;17:664–670. 19634210 10.1097/jgp.0b013e3181a6516e

[4] Launer LJ, White LR, Petrovitch H, Ross GW, Curb JD. Cholesterol and neuropathologic markers of AD: a population-based autopsy study. Neurology 2001;57:1447–1452. 11673587 10.1212/wnl.57.8.1447

[5] Matsuzaki T, Sasaki K, Hata J, et al. Association of Alzheimer disease pathology with abnormal lipid metabolism: the Hisayama Study. Neurology 2011;77:1068–1075. 21911734 10.1212/WNL.0b013e31822e145d

[6] Dubois B, Hampel H, Feldman HH, et al. Preclinical Alzheimer's disease: definition, natural history, and diagnostic criteria. Alzheimers Dement 2016;12:292–323. 27012484 10.1016/j.jalz.2016.02.002 PMC6417794

[7] Blennow K, Mattsson N, Scholl M, Hansson O, Zetterberg H. Amyloid biomarkers in Alzheimer's disease. Trends Pharmacol Sci 2015;36:297–309. 25840462 10.1016/j.tips.2015.03.002

[8] Choi HJ, Byun MS, Yi D, et al. Association between serum triglycerides and cerebral amyloidosis in cognitively normal elderly. Am J Geriatr Psychiatry 2016;24:604–612. 27311886 10.1016/j.jagp.2016.03.001

[9] Gottesman RF, Schneider AL, Zhou Y, et al. Association between midlife vascular risk factors and estimated brain amyloid deposition. JAMA 2017;317:1443–1450. 28399252 10.1001/jama.2017.3090 PMC5921896

[10] Vemuri P, Knopman DS, Lesnick TG, et al. Evaluation of amyloid protective factors and Alzheimer disease neurodegeneration protective factors in elderly individuals. JAMA Neurol 2017;74:718–726. 28418521 10.1001/jamaneurol.2017.0244 PMC5649401

[11] Schaefer EJ, Tsunoda F, Diffenderfer M, Polisecki E, Thai N, Asztalos B. The Measurement of Lipids, Lipoproteins, Apolipoproteins, Fatty Acids, and Sterols, and Next Generation Sequencing for the Diagnosis and Treatment of Lipid Disorders. South Dartmouth, MA: Endotext; 2000. 27099900

[12] Kontush A. HDL particle number and size as predictors of cardiovascular disease. Front Pharmacol 2015;6:218. 26500551 10.3389/fphar.2015.00218 PMC4593254

[13] Musunuru K, Orho-Melander M, Caulfield MP, et al. Ion mobility analysis of lipoprotein subfractions identifies three independent axes of cardiovascular risk. Arterioscler Thromb Vasc Biol 2009;29:1975–1980. 19729614 10.1161/ATVBAHA.109.190405 PMC2772123

[14] Mora S, Caulfield MP, Wohlgemuth J, et al. Atherogenic lipoprotein subfractions determined by ion mobility and first cardiovascular events after random allocation to high-intensity statin or placebo the justification for the use of statins in prevention: an intervention trial evaluating rosuvastatin (JUPITER) trial. Circulation 2015;132:2220–2229. 26408274 10.1161/CIRCULATIONAHA.115.016857 PMC4674425

[15] Berglund G, Elmstahl S, Janzon L, Larsson SA. The Malmo Diet and Cancer Study: design and feasibility. J Intern Med 1993;233:45–51. 8429286 10.1111/j.1365-2796.1993.tb00647.x

[16] Manjer J, Carlsson S, Elmstahl S, et al. The Malmo Diet and Cancer Study: representativity, cancer incidence and mortality in participants and non-participants. Eur J Cancer Prev 2001;10:489–499. 11916347 10.1097/00008469-200112000-00003

[17] Rosvall M, Persson M, Ostling G, et al. Risk factors for the progression of carotid intima-media thickness over a 16-year follow-up period: the Malmo Diet and Cancer Study. Atherosclerosis 2015;239:615–621. 25746169 10.1016/j.atherosclerosis.2015.01.030

[18] Hedblad B, Nilsson P, Janzon L, Berglund G. Relation between insulin resistance and carotid intima-media thickness and stenosis in non-diabetic subjects: results from a cross-sectional study in Malmo, Sweden. Diabet Med 2000;17:299–307. 10821297 10.1046/j.1464-5491.2000.00280.x

[19] Gustavsson AM, Stomrud E, Abul-Kasim K, et al. Cerebral microbleeds and white matter hyperintensities in cognitively healthy elderly: a cross-sectional cohort study evaluating the effect of arterial stiffness. Cerebrovasc Dis Extra 2015;5:41–51. 26120319 10.1159/000377710 PMC4478329

[20] Blennow K, Hampel H, Weiner M, Zetterberg H. Cerebrospinal fluid and plasma biomarkers in Alzheimer disease. Nat Rev Neurol 2010;6:131–144. 20157306 10.1038/nrneurol.2010.4

[21] Palmqvist S, Zetterberg H, Blennow K, et al. Accuracy of brain amyloid detection in clinical practice using cerebrospinal fluid beta-amyloid 42: a cross-validation study against amyloid positron emission tomography. JAMA Neurol 2014;71:1282–1289. 25155658 10.1001/jamaneurol.2014.1358

[22] Koole M, Lewis DM, Buckley C, et al. Whole-body biodistribution and radiation dosimetry of 18F-GE067: a radioligand for in vivo brain amyloid imaging. J Nucl Med 2009;50:818–822. 19372469 10.2967/jnumed.108.060756

[23] Lundqvist R, Lilja J, Thomas BA, et al. Implementation and validation of an adaptive template registration method for 18F-flutemetamol imaging data. J Nucl Med 2013;54:1472–1478. 23740104 10.2967/jnumed.112.115006

[24] Schmidt P, Gaser C, Arsic M, et al. An automated tool for detection of FLAIR-hyperintense white-matter lesions in multiple sclerosis. Neuroimage 2012;59:3774–3783. 22119648 10.1016/j.neuroimage.2011.11.032

[25] Caulfield MP, Li S, Lee G, et al. Direct determination of lipoprotein particle sizes and concentrations by ion mobility analysis. Clin Chem 2008;54:1307–1316. 18515257 10.1373/clinchem.2007.100586

[26] Qiu C, Xu W, Fratiglioni L. Vascular and psychosocial factors in Alzheimer's disease: epidemiological evidence toward intervention. J Alzheimers Dis 2010;20:689–697. 20182015 10.3233/JAD-2010-091663

[27] O'Leary DH, Bots ML. Imaging of atherosclerosis: carotid intima-media thickness. Eur Heart J 2010;31:1682–1689. 20542989 10.1093/eurheartj/ehq185

[28] Hughes TM, Lopez OL, Evans RW, et al. Markers of cholesterol transport are associated with amyloid deposition in the brain. Neurobiol Aging 2014;35:802–807. 24199960 10.1016/j.neurobiolaging.2013.09.040 PMC3896052

[29] Reed B, Villeneuve S, Mack W, DeCarli C, Chui HC, Jagust W. Associations between serum cholesterol levels and cerebral amyloidosis. JAMA Neurol 2014;71:195–200. 24378418 10.1001/jamaneurol.2013.5390 PMC4083819

[30] Toledo JB, Toledo E, Weiner MW, et al. Cardiovascular risk factors, cortisol, and amyloid-beta deposition in Alzheimer's Disease Neuroimaging Initiative. Alzheimers Demen 2012;8:483–489. 10.1016/j.jalz.2011.08.008 PMC3668456 23102118

[31] Kivipelto M, Helkala EL, Laakso MP, et al. Apolipoprotein E epsilon4 allele, elevated midlife total cholesterol level, and high midlife systolic blood pressure are independent risk factors for late-life Alzheimer disease. Ann Intern Med 2002;137:149–155. 12160362 10.7326/0003-4819-137-3-200208060-00006

[32] Solomon A, Kivipelto M, Wolozin B, Zhou J, Whitmer RA. Midlife serum cholesterol and increased risk of Alzheimer's and vascular dementia three decades later. Dement Geriatr Cogn Disord 2009;28:75–80. 19648749 10.1159/000231980 PMC2814023

[33] Whitmer RA, Sidney S, Selby J, Johnston SC, Yaffe K. Midlife cardiovascular risk factors and risk of dementia in late life. Neurology 2005;64:277–281. 15668425 10.1212/01.WNL.0000149519.47454.F2

[34] Di Paolo G, Kim TW. Linking lipids to Alzheimer's disease: cholesterol and beyond. Nat Rev Neurosci 2011;12:284–296. 21448224 10.1038/nrn3012 PMC3321383

[35] Burgess BL, McIsaac SA, Naus KE, et al. Elevated plasma triglyceride levels precede amyloid deposition in Alzheimer's disease mouse models with abundant A beta in plasma. Neurobiol Dis 2006;24:114–127. 16899370 10.1016/j.nbd.2006.06.007

[36] Araki W, Tamaoka A. Amyloid beta-protein and lipid rafts: focused on biogenesis and catabolism. Front Biosci 2015;20:314–324. 10.2741/4311 25553453

[37] Hellstrand E, Sparr E, Linse S. Retardation of Abeta fibril formation by phospholipid vesicles depends on membrane phase behavior. Biophys J 2010;98:2206–2214. 20483329 10.1016/j.bpj.2010.01.063 PMC2872260

[38] Mamo JC, Jian L, James AP, Flicker L, Esselmann H, Wiltfang J. Plasma lipoprotein beta-amyloid in subjects with Alzheimer's disease or mild cognitive impairment. Ann Clin Biochem 2008;45:395–403. 18583625 10.1258/acb.2008.007214

[39] Agrawal N, Freitas Corradi P, Gumaste N, Goldberg IJ. Triglyceride treatment in the age of cholesterol reduction. Prog Cardiovasc Dis 2016;59:107–118. 27544319 10.1016/j.pcad.2016.08.003 PMC5364728

[40] Berglund L, Brunzell JD, Goldberg AC, Goldberg IJ, Stalenhoef A. Treatment options for hypertriglyceridemia: from risk reduction to pancreatitis. Best Pract Res Clin Endocrinol Metab 2014;28:423–437. 24840268 10.1016/j.beem.2013.10.002 PMC4028601

